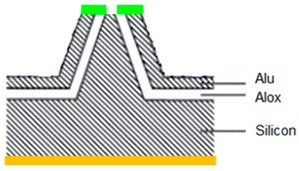# Advanced Surface Probing Using a Dual-Mode NSOM–AFM Silicon-Based Photosensor

**DOI:** 10.3390/nano9121792

**Published:** 2019-12-16

**Authors:** Matityahu Karelits, Emanuel Lozitsky, Avraham Chelly, Zeev Zalevsky, Avi Karsenty

**Affiliations:** 1 Advanced Laboratory of Electro-Optics (ALEO), Department of Applied Physics/Electro-Optics Engineering, Lev Academic Center, Jerusalem 9116001, Israel; matityahukarelits@gmail.com (M.K.); lozitsky@g.jct.ac.il (E.L.); 2 Faculty of Engineering, Bar-Ilan University, Ramat Gan 5290002, Israel; chellyr@gmail.com (A.C.); Zeev.Zalevsky@biu.ac.il (Z.Z.); 3 The Nanotechnology Center, Bar-Ilan University, Ramat Gan 5290002, Israel; 4 The Nanotechnology Center for Education and Research, Lev Academic Center, Jerusalem 9116001, Israel

**Keywords:** near-field scanning optical microscope (NSOM), atomic force microscope (AFM), dual-mode, silicon, photodetector, pinhole subwavelength aperture, signal-to-noise ratio (SNR)

## Abstract

A feasibility analysis is performed for the development and integration of a near-field scanning optical microscope (NSOM) tip–photodetector operating in the visible wavelength domain of an atomic force microscope (AFM) cantilever, involving simulation, processing, and measurement. The new tip–photodetector consists of a platinum–silicon truncated conical photodetector sharing a subwavelength aperture, and processing uses advanced nanotechnology tools on a commercial silicon cantilever. Such a combined device enables a dual-mode usage of both AFM and NSOM measurements when collecting the reflected light directly from the scanned surface, while having a more efficient light collection process. In addition to its quite simple fabrication process, it is demonstrated that the AFM tip on which the photodetector is processed remains operational (i.e., the AFM imaging capability is not altered by the process). The AFM–NSOM capability of the processed tip is presented, and preliminary results show that AFM capability is not significantly affected and there is an improvement in surface characterization in the scanning proof of concept.

## 1. Introduction

### 1.1. Surface Scanning Background and Needs

On one hand, atomic force microscopy (AFM) is the core method of scanning probe microscopy (SPM) enabling nanometric surface morphology characterization. On the other hand, near-field scanning optical microscopy (NSOM) is an optical method for subdiffractive optical characterization, which could be a complementary analysis to the AFM technique in the optical domain, for instance for characterizing fluorescence or electroluminescence of nanoscale structures. NSOM has remained one of the most challenging optics domains since it was discovered three decades ago [1]. The concepts of NSOM and Scanning Near-field Optical Microscopy (SNOM) date from the beginning of the 20th century. However, the first experimental proof was presented by Pohl [2]. Both AFM and NSOM methods are crucial for many fields of science, technology, and industry, and are usually used separately [3,4,5]. These complementary methods are widely used for nanoscale study and characterization of new nanomaterial components, life science and biological objects [6], semiconductor and electronic metrology, and in photonics and plasmonics [7]. Several NSOM techniques have been proposed, such as differential NSOM [8], active-tip NSOM [9,10], and more. Mapping of the light detection at subwavelength scales in nanophotonic structures and techniques has been reviewed previously [11]. To date, there are less than twenty key players and manufacturers in the scanning probe microscopy (SPM) market worldwide [12,13], with some of them playing leading roles in AFM [14] and NSOM [15] technologies. The growing need to conduct research studies on various biological and nonbiological aspects to develop innovative solutions has led manufacturers to seek equipment that integrates leading technological features. In addition, demand for technologically equipped microscopes in various educational institutions continues to increase, which is attributed to the growing need for in-depth information and knowledge. The surge in the production of various electronic products has led to increasing demand for atomic force microscopes in the global market.

### 1.2. AFM–NSOM Dual-Mode Concept

**Figure 1:**
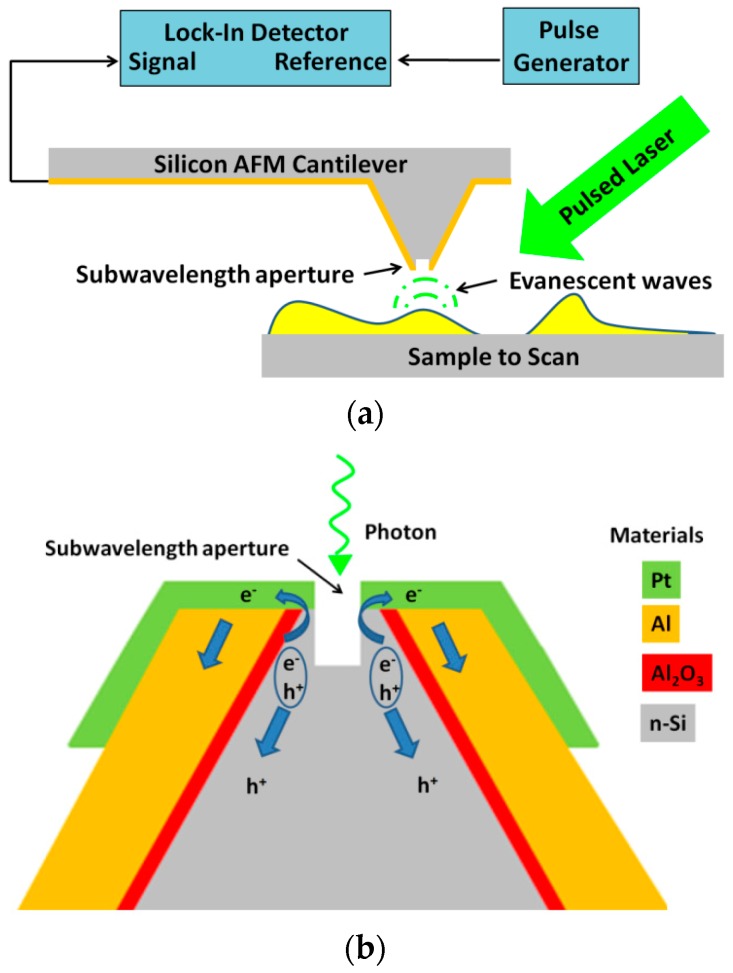
Schematic representation of the atomic force microscope combined with the near-field scanning optical microscope (AFM–NSOM) dual-mode proposed system: (**a**) Concept of the AFM–NSOM coupled device; (**b**) Concept of the processed tip structure and photocurrent generation.

AFM and NSOM techniques have progressed in parallel in recent decades, with each one providing advantages. Moreover, significant progress was recently observed in the manufacture of new tips using processing methods [16,17], and the simulation of new tip designs for future manufacture [18]. Case studies combining organic light-emitting devices (OLEDs) with micromachined silicon cantilevers [19], as well as organic photodetectors [20], were also investigated. The idea of combining the two measurement capabilities, AFM and NSOM, into one dual mode based on silicon tips is quite unique and presents several advantages. The main important advantages that the proposed concept offers include the following features: having dual modes in one tip, use of standard commercial starting materials, multifunctionality, energetic efficiency, light acquisition at the surface of the sample, and good signal-to-noise ratio (SNR). The AFM–NSOM dual-mode improved system is presented in Figure 1. The truncated photodetector is used instead of a regular silicon tip, which is placed at the end of the cantilever, creating a reproducible photosensor integrated into an AFM tip, as described later. Some earlier works on combined AFM–NSOM or AFM–SNOM were reported in the past [21,22], which presented some advantages. However, those configurations are admittedly not the same (i.e., do not work with a Schottky diode), and additional novel features are presented in the design of our new device. In an earlier but different concept was also investigated [23], where a Schottky diode was realized on a SNOM, however the concept was totally different from the proposed work (Figure 1).

### 1.3. Multifunctionality and Energetic Efficiency

In the system, NSOM and AFM functionalities are integrated in the same tip. The AFM functionality can be adjusted by properly designing the mechanical responsivity of the tip. The NSOM functionality of near-field imaging can be obtained according to the size of the detector on the tip, its responsivity, and its distance from the inspected surface. Since the collected evanescent waves are converted to an electrical read-out directly on the tip, and do not need to be coupled to the fiber or be guided to a remote detector, one can expect a higher signal, higher SNR, and eventually higher resolution (as resolution is directly related to SNR). 

As previously proven in large number of publications and in the basic theory of diffraction, the illumination field consists of both harmonic and evanescent components, with μ being the spatial frequency and λ the optical wavelength; for μ > 1/λ we have evanescent waves and for μ < 1/λ harmonic waves. The efficiency of the probe will be composed of both. The harmonic component of the electrical field is reflected on the coupling efficiency into the fiber tip in the following manner:

- Geometric efficiency:

(1) $$\eta_{geo}=\frac{A_{tip}}{A_{scatterer}}$$

where $A_{tip}$ is the light collecting area of the tip and $A_{scatterer}$ is the area of the scatterer from which the light is back scattered, and which we aim to collect in the tip.

- Angular efficiency:

(2) $$\eta_{angle}=\frac{NA^{2}}{\theta_{scatterer}{}^{2}}$$

where *NA* is the Numerical aperture of the fiber and $\theta_{scatterer}$ is the angular scattering of the scatterer. This angle is proportional to:

(3) $$\theta_{scatterer}{}^{2}\propto \frac{\lambda^{2}}{A_{scatterer}}$$

where λ is the wavelength, since we try to sense subwavelength spatial features $\theta_{scatterer}$ approaching a hemisphere.

- Fresnel efficiency:

The refraction index of the fiber tip is different from the medium in which the back scatterer radiation propagates. The power reflectance at normal incidence is then given by:

(4) $$R={\left(\frac{n_{fiber}-1}{n_{fiber}+1}\right)}^{2}$$

where $n_{fiber}$ is the refraction index of the fiber’s tip, and the Fresnel factor for coupling efficiency is approximately equal to:

(5) $$\eta_{Fresnel}=1-R$$

The overall efficiency of the light collection process equals to:

(6) $$\eta_{total}=\eta_{geo\;}\eta_{angle\;}\eta_{Fresnel}$$

Note that the evanescent component of the electrical field has similar expressions for the collection efficiency, however, the intensity of the electrical field that interacts with the tip will be exponentially decayed with the distance between the scatterer and the tip:

(7) $$I_{y}\left(\mu ,z\right)=I_{y}\left(\mu ,0\right)exp\left(-4\pi \sqrt{\mu^{2}-\frac{1}{\lambda^{2}}}\right)z$$

with μ > 1/λ for the evanescent component. This decaying reduction in power needs to be taken into account as well, since less energy arrives at the tip, and thus less is collected.

Therefore, the efficiency in coupling the read-out light to the NSOM fiber tip and guiding it backwards to a remote detector depends on the cross-section of the fiber’s mode (the guided area) and its *NA*, as well as the Fresnel coefficient. A detector fabricated on the tip can have an *NA* value much larger than that of the fiber tip, while also reaching a hemisphere and being equal to the *NA* value of the scatterer itself. The detector can have an antireflective coating that reduces the Fresnel coefficient *R* to zero, causing $\eta_{Fresnel}$ to equal one, while in the fiber tip it is very difficult to fabricate an antireflective coating on the small area and not the flat tip. Thus, the main limit to the read-out efficiency in the proposed approach, which is based on direct light collection with a detector, is related only to the area of the detector. In addition, since the detector senses only intensity and not the field, the coupling efficiency is much less dependent on the relative angle and the relative orientation between the light-collecting tip and the location of the sample from which the light is being collected. In our case, the sensing is done directly on the evanescent waves (which are converted into electrons in the detector positioned at the edge of the tip), while for the back-guiding conventional NSOM tip, this includes the conversion of the evanescent waves to guided modes being delivered to a remote detector positioned on the other side of the NSOM fiber. This conversion and delivery efficiency (guiding losses of the NSOM fiber) is also far from 100%, even if the areas and the angular range (*NA*) of the tip are well-matched to the scatterer. Note that the overlapping integral mode is defined as:

(8) $$\eta =\frac{{\left(\iint \Phi_{tip}\left(x,y\right)\Phi_{eva\_mode}{}^{*}\left(x,y\right)dxdy\right)}^{2}}{\left(\iint \Phi_{tip}\left(x,y\right)\Phi_{tip}{}^{*}\left(x,y\right)dxdy\right)\left(\iint \Phi_{eva\_mode}\left(x,y\right)\Phi_{eva\_mode}{}^{*}\left(x,y\right)dxdy\right)}$$

where $\Phi_{\mathrm{tip}}\left(x,y\right)$ is the 2-D mode supported by the fiber tip and $\Phi_{\mathrm{eva}\_\mathrm{mode}}\left(x,y\right)$ is the 2-D evanescent mode propagated from the scatterer towards the fiber tip. In the case of a detector fabricated on the surface of the tip, one does not need to couple the light and guide it back to a remote detector, and thus the mode conversion efficiency is maximal and no guiding losses are exhibited.

## 2. Materials and Methods

### 2.1. Finite Elements Method (FEM)

The Comsol Multi-Physics software package [24], based on the finite elements method (FEM) [25,26], was employed to perform the numerical study of the proposed device. The device consists of a silicon Schottky photodiode bearing a subwavelength top aperture [27]. Such a nanoscale electro-optical sensor placed on an AFM cantilever’s edge ensures collection of the topography and the optical data. The electric response while scanning a laser beam was studied and optimized by changing the related specification. It was shown that high resolution of order of the detector’s aperture was obtained [27]. This is useful for validation of the behavior of the device in comparison to current standards and benchmarks. As a first step, the tip–photodetector was simulated separately, then as a second step, it was combined with a simulated standard silicon-based cantilever. 

### 2.2. Simulations Results of the Detector Structure

**Figure 2:**
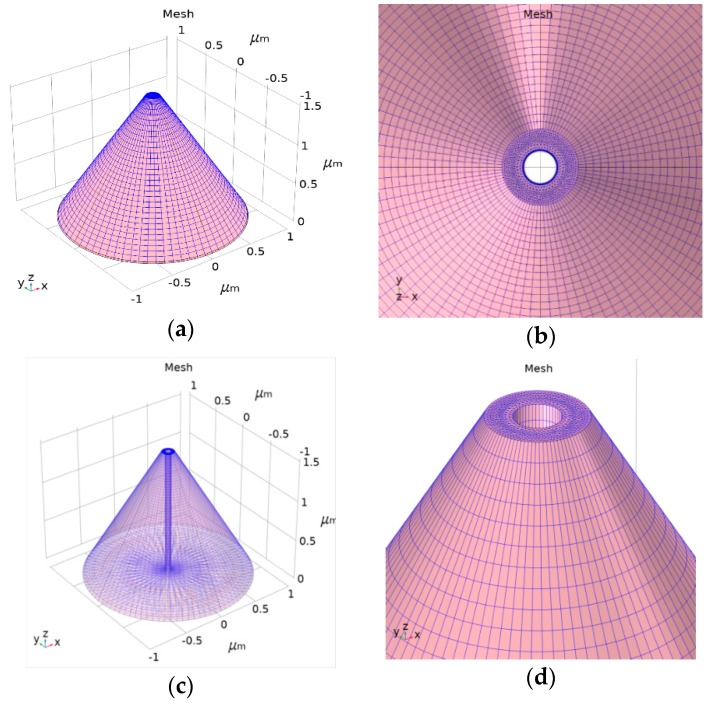
Comsol structure and mesh simulation results of the tip–photodetector. Its main parameters are: height of 1.6 µm, top diameter of 150 nm, and bottom radius less than 1 µm: (**a**) simulated regular mesh used for external contacts; (**b**) simulated accurate mesh used for upper inside aperture; (**c**) simulated accurate mesh used for internal drilled cylinder of the photodetector; (**d**) zoom-in of the aperture, sharing a diameter of 150 nm only less than the visible wavelength, and of the internal drilled cylinder.

**Figure 3:**
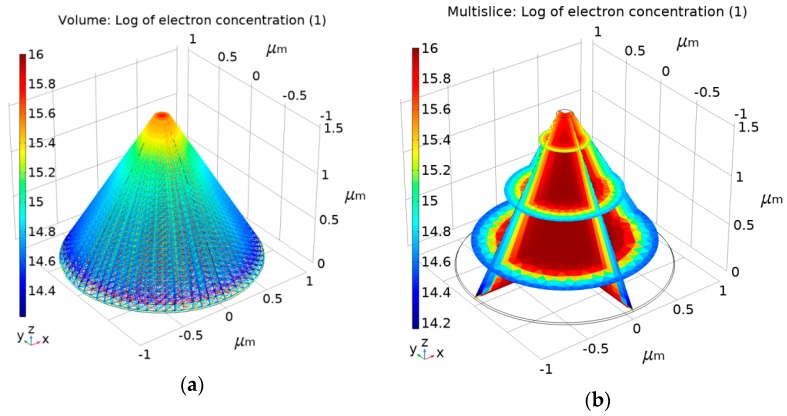
Comsol electrical simulation results of the tip–photodetector at equilibrium (300 K): (**a**) volume log of the electron concentration (cm^−3^ units); (**b**) multislice log of the electron concentration (cm^−3^ units).

Such a nanoscale electro-optical sensor placed on an AFM cantilever’s edge ensures collection of the topography and the optical data. As shown in Figure 2 and Figure 3, the NSOM is a 3-D structure with a truncated, conical-shaped photodetector device. Its main parameters are: height of 1.6 μm, top radius of 75 nm, and bottom radius less than 1 μm. For simulation considerations, such as mesh complexity and run time, the height of 1.6 μm considered here is 1/10 of the real AFM silicon tip height. Figure 3 presents the Comsol electrical preliminary simulation results using the semiconductor module. This prefunctionality simulation was performed under the following conditions: equilibrium (300 K) and reverse bias of 0.5 V applied at the upper half of the tip as a Schottky contact and grounded at the bottom as an Ohmic contact; n doping was 10^16^ cm^−3^; and the work function was 4.72 eV (Al). Figure 3a presents the volume log of the electron concentration (cm^−3^ units), and Figure 3b presents the multislice log of the electron concentration (cm^−3^ units).

## 3. Results

### 3.1. Six-Step Quick Process Flow Overview

One of the main advantages of the AFM–NSOM dual-mode photodetector concept is the fact that the fabrication process is quite simple, short (a couple of hours overall), and starts from a commercial AFM silicon tip. As presented in Table 1, the full process is performed in six steps: (1) preparation of standard commercial (FESP-V2 model from Bruker, Karlsruhe, Germany) [28] AFM silicon-based tips (n^+^ type, As < 10^17^ cm^−3^); (2) atomic layer deposition (ALD) of a passivation layer (Al_2_O_3_, 50 nm thick); (3) sputtering deposition of reflective and conductive layer (Al, 200 nm thick); (4) Focused Ion Beam (FIB) ablation of the tip to get the initial silicon aperture size (100 nm diameter); (5) in situ FIB deposition of platinum thick opaque layer of 100 nm; and (6) in situ FIB drilling to define the final silicon aperture (<100 nm diameter). 

Even though the described process is short and reproducible, from a long-term perspective, the ultimate goal would be to propose an enhanced process in which such special probes are stably mass-produced as an industrial application. There are currently few alternative directions that are checked in parallel. However, in this first publication, the focus is on the feasibility analysis of a newly designed tip built from commercial cantilevers.

**Figure 4:**
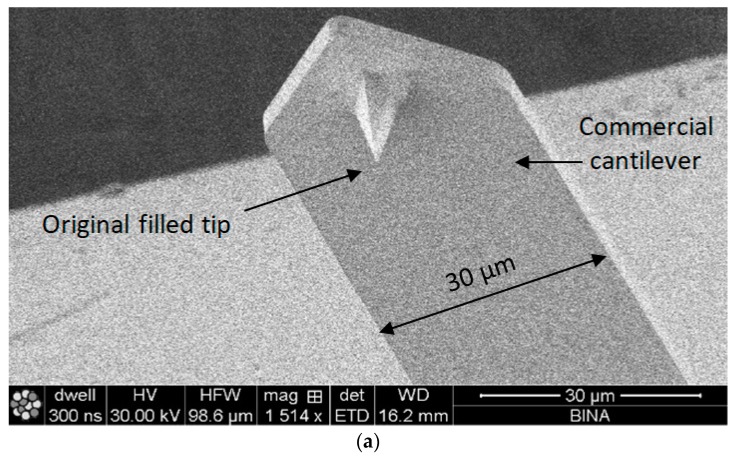

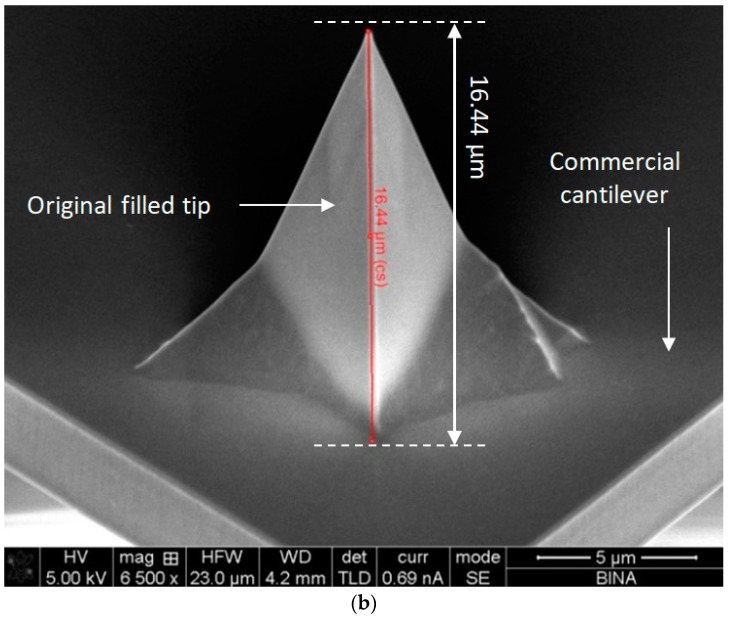
Scanning electron microscopy (SEM) pictures of the AFM cantilever commercial starting material: (**a**) downside view of the cantilever and of the tip; (**b**) upside and close-up views of the processed tip. (HV—High Voltage; HFW—Horizontal Field Width; WD—Working Distance; SE—Secondary Electrons; BINA—Bar Ilan Institute for Nanotechnology and Advanced materials)

The profile and dimensions of the commercial cantilever and of the commercial tip are presented in Figure 4a,b, respectively. 

### 3.2. Consecutive Focus Ion Beam (FIB) Steps

**Figure 5:**
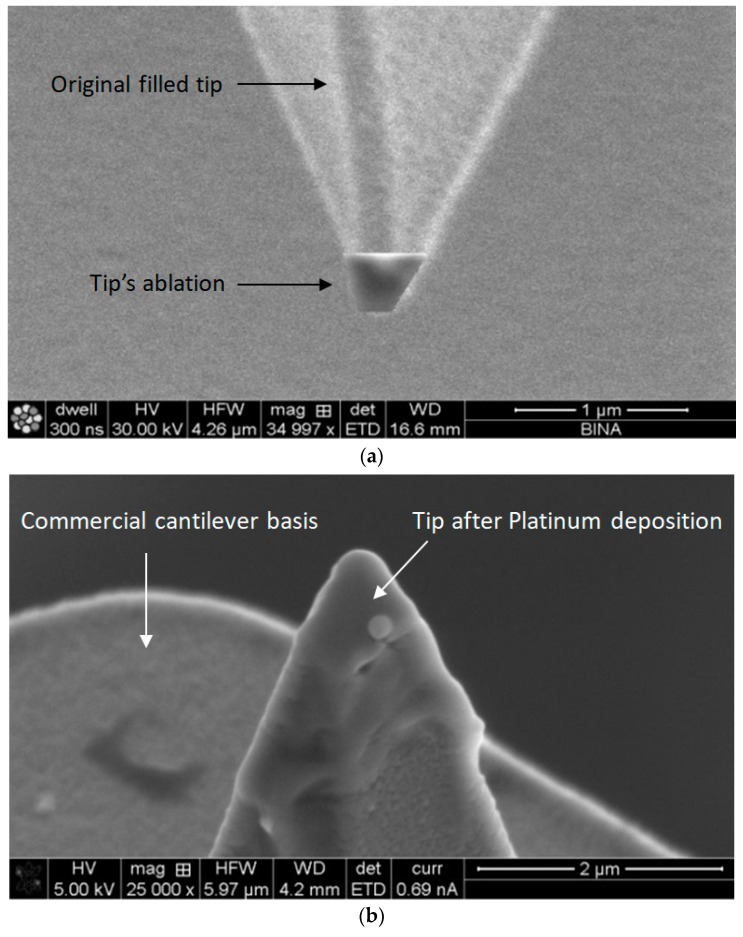
SEM view of the tip area using the FIB system: (**a**) after tip ablation: (**b**) after platinum deposition.

The next three focused ion beam (FIB) steps—tip ablation, platinum deposition, and tip drilling—were processed using the Field Electron and Ion company (FEI) Helios 600 system, which is a dual-beam instrument combining scanning electron microscopy (SEM) and FIB technologies, as well as gas chemistries, different detectors, and manipulators. The tip ablation was performed under the following conditions: the tip was cut with a Ga+ ion beam current of 2.7 nA, accelerated by a voltage of 2 kV fixed on the FIB device (Figure 5a), and the platinum from the vapor precursor was deposited by the Ga+ ion beam on the top of the probe (Figure 5b). The final step was drilling the aperture hole of the detector. This was performed with a Ga+ ion beam (9.7 pA, 30 kV). The depth of the drilling was 400 nm through the platinum layer, and a conical hole was obtained. Regarding the concerns and considerations in the choice of the FIB, it was important to pay attention to some critical parameters. There were two types of FIB in the nanocenter: the first one used Ga+ ions, while the second one used He ions. For this process, Ga+ ions are necessary, since the He is not strong enough to enable a truncated tip. It is known that during FIB machining, Ga+ ions are implanted into the Si structure. These implants can also be located in the critical area where there is a need to detect a photo current. Such a situation can influence the SNR. Moreover, when compared to the implantation process for regular ions, there is no annealing step or any thermal post-recovery process after the drilling stage. The main concern would be that the Ga+ ions can cause degradation of the initial silicon crystalline structure, and as a consequence, can affect the electro-optic measurements. Even so, the quality of the electrode and the Schottky contact were checked and were found to be very good. The presented process (Table 1) is also reproducible.

**Figure 6:**
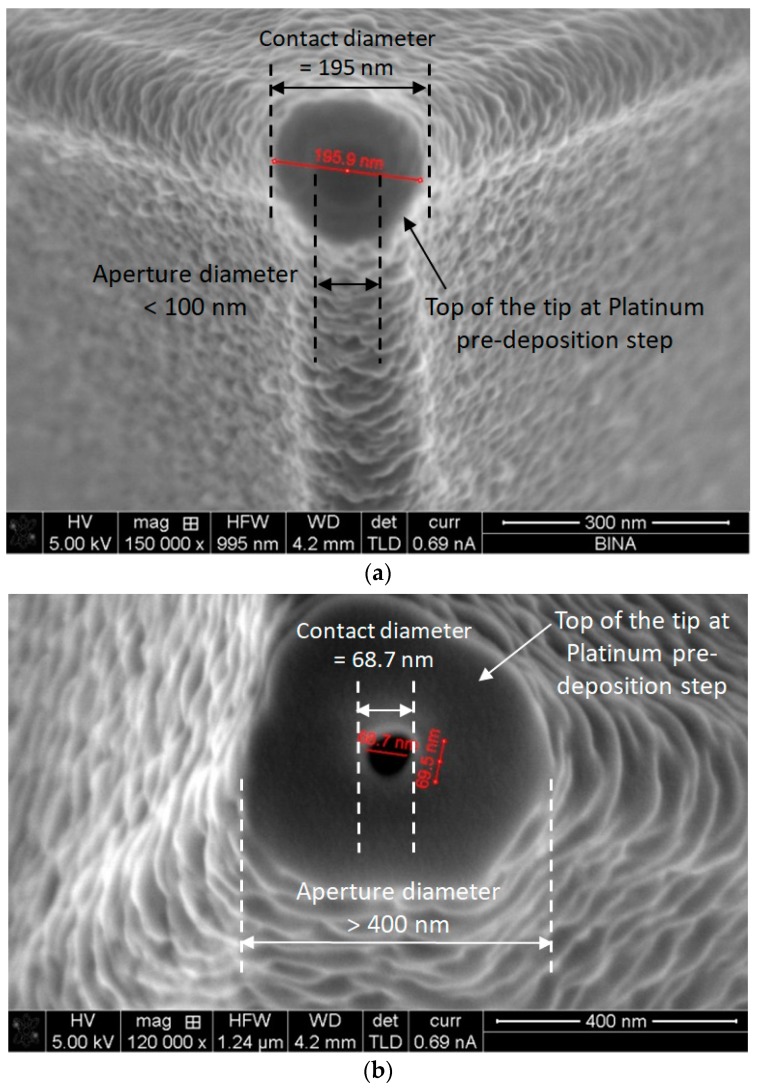
SEM pictures of two AFM tips after drilling: (**a**) preplatinum deposition view of a tip with a contact diameter of 195 nm and aperture diameter of 100 nm; (**b**) postplatinum deposition view of a tip with a contact diameter of ~400 nm and an aperture diameter of 68.7 nm.

As shown in Figure 6, a series of three tips was processed while varying the diameters in the range of tens of nanometers. The drilling of the ablated tip using the FIB system is the critical part of the process. Two important parameters should be set. The first one is the diameter of the drilled aperture, which varies from 68.7 nm (Figure 6a) to 49.8 nm (Figure 6b). The second parameter is the contact surface or contact diameter. The reason is that we may conserve the AFM capability of scanning, even if we add the NSOM capability of light collection. The standard contact radius of the AFM tip is less than 10 nm [28]. In the first experiences, the obtained contact diameter of the tip was about 400 nm (Figure 6a). The next experiences and ameliorations enabled a reduced contact diameter of less than 195 nm (Figure 6b), which was ten times larger than the commercial tip’s contact diameter. In spite of the larger contact diameter, this tip can still function as an AFM scanning tip; however, the resolution may be affected. It appears that there is a trade-off between the two important diameters, namely drill and contact diameters, and we may look for the optimal dimensions in the future. 

### 3.3. AFM Scan Functionality Check

**Figure 7:**
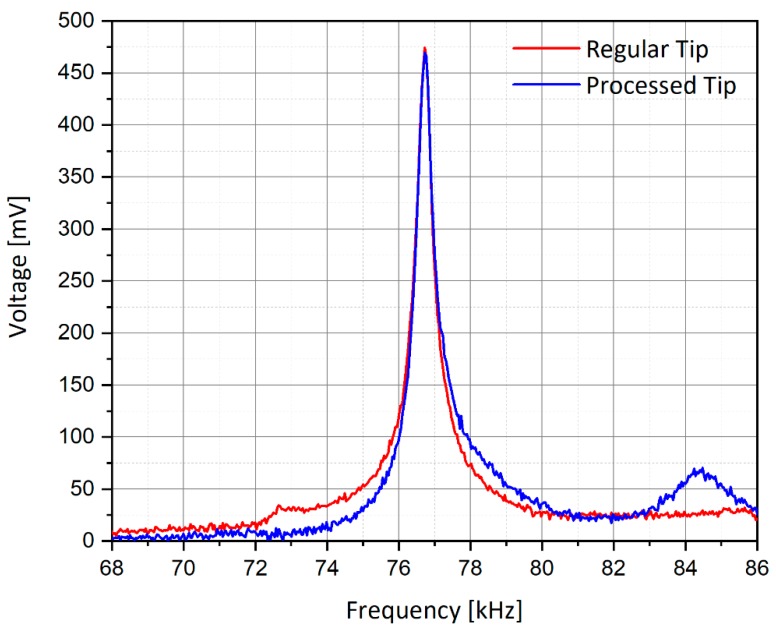
Piezoelectric resonance curves of the regular and processed tips as a function of the excitation frequency of the AFM system.

**Figure 8:**
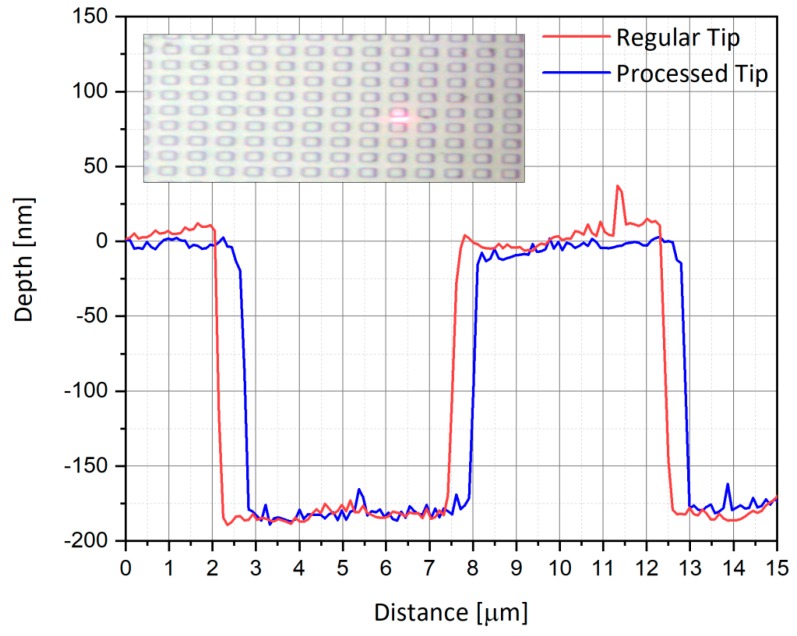
Comparison of the AFM scans of the calibration sample for the reference tip and the processed tip. The structure has dimensions of 10 µm pitch and 180 nm height, and is well resolved by both tips, as shown in the insert image. The visible laser spot in the insert is used as the AFM cantilever guide.

After the processing step, it was important to assure the AFM functionality of the processed tip. To do so, a calibration sample was used, sharing a periodical structure made of gold rectangular wells of 10 µm pitch and 180 nm step depth. For this purpose, a scan was performed using a reference (initially unprocessed) tip and compared with the processed tip of the probe. The main test was to check that the tip resonance was not dramatically modified by the process, as shown in Figure 7, and the scan of the calibration sample was similar for the two tips (Figure 8). Indeed, one can observe that the same 2-D *X*-*Y* resolution and *Z*-resolution was obtained despite the increased weight of the new probe. Usually, regular tips respond to the AFM piezoelectric system occurring at a certain resonance frequency, enabling controlled movements. This kind of response was obtained for a reference tip and is presented in Figure 7. After identifying the correct resonance, the obtained scanning was successful, and a step function of the sample was obtained (shown in Figure 8). An optical microscope view of the calibration sample to be scanned with the regular and processed AFM tips is shown in the enclosed view of Figure 8. Therefore, the AFM usage (model Bruker AXS, Bio FastScan) of the proposed tip was proven. Horizontal and vertical accuracies are a function of the tip’s geometry.

### 3.4. AFM Grating Check

As part of the investigation regarding the accuracy of the AFM scanning measurements to be performed with processed vs. regular tips, it was necessary to check the grating test. This check includes a sample made of an array of very sharp silicon tips [29]. The grating itself is built on a silicon wafer top surface, and the tips are very sharp. The test grating is intended for 3-D visualization of the scanning tip, serving as a mirror. Also, the test enables the determination of tip sharpness parameters (aspect ratio), tip degradation, and contamination control. 

**Figure 9:**
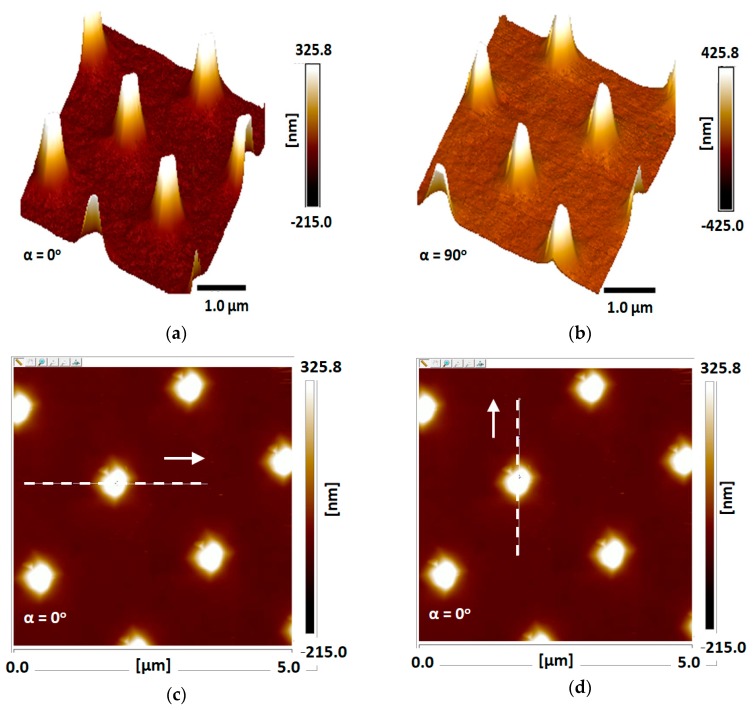

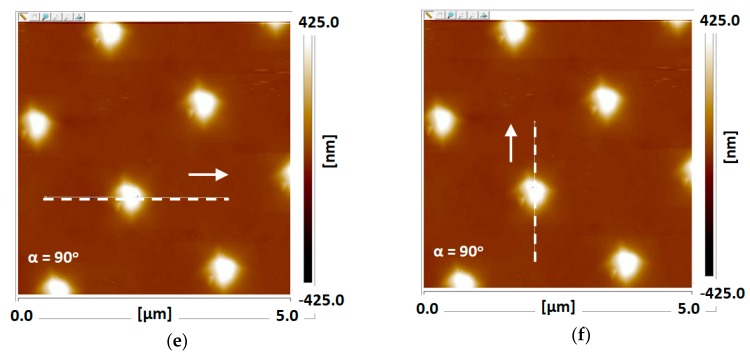
Advanced AFM scans of nano-Si tips using a regular AFM tip: (**a**) prescan sample at α = 0°; (**b**) prescan sample rotated at α = 90°; (**c**) horizontal scan view at α = 0°; (**d**) vertical scan view at α = 0°; (**e**) horizontal scan view at α = 90°; (**f**) vertical scan view at α = 90°.

**Figure 10:**
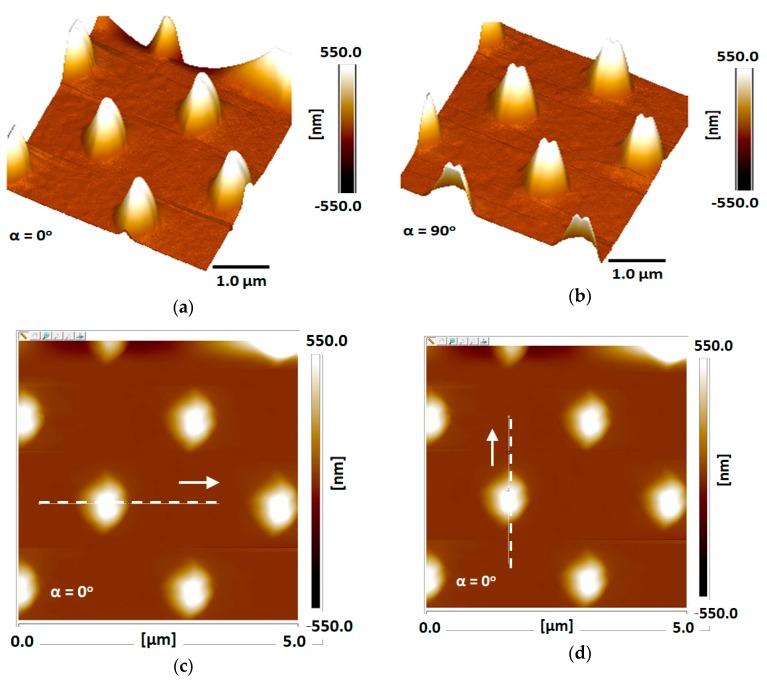

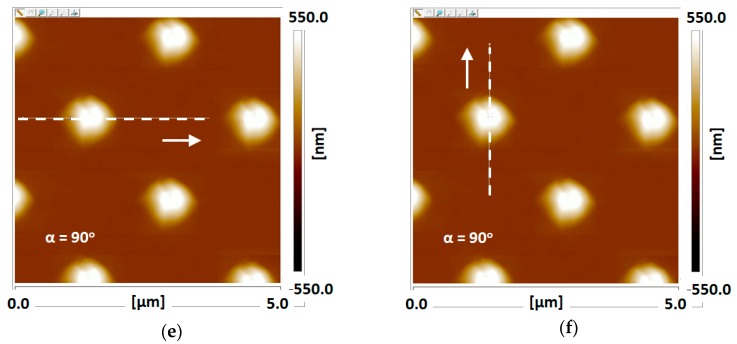
Advanced AFM scans of nano-Si tips using processed tip: (**a**) prescan sample at α = 0°; (**b**) prescan sample rotated at β = 90°; (**c**) horizontal scan view at α = 0°; (**d**) vertical scan view at α = 0°; (**e**) horizontal scan view at β = 90°; (**f**) vertical scan view at β = 90°.

Following these aims, both the regular AFM tip (Figure 9) and the processed one (Figure 10) were used to scan the array in complementary tests. The regular tip was checked in horizontal (Figure 9c,e) and vertical (Figure 9d,f) directions, while also changing the angle of the scanning from α = 0° (Figure 9c,d) to α = 90° (Figure 9e,f). The same scanning checks were performed for the processed tip in the horizontal (Figure 10c,e) and vertical (Figure 10d,f) directions, while changing the angle of the scanning from α = 0° (Figure 10c,d) to α = 90° (Figure 10e,f). 

**Figure 11:**
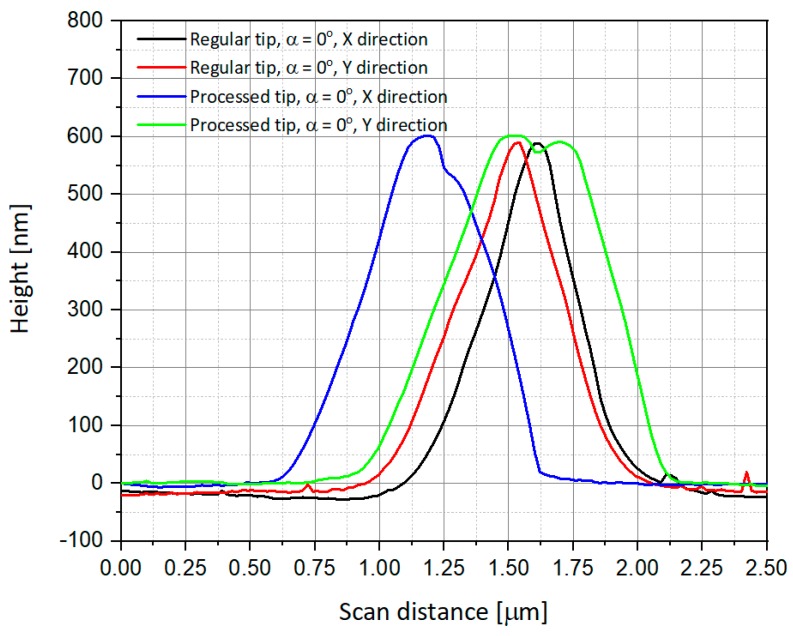
Comparison of the scanning using AFM regular and processed tips for α = 0°.

**Figure 12:**
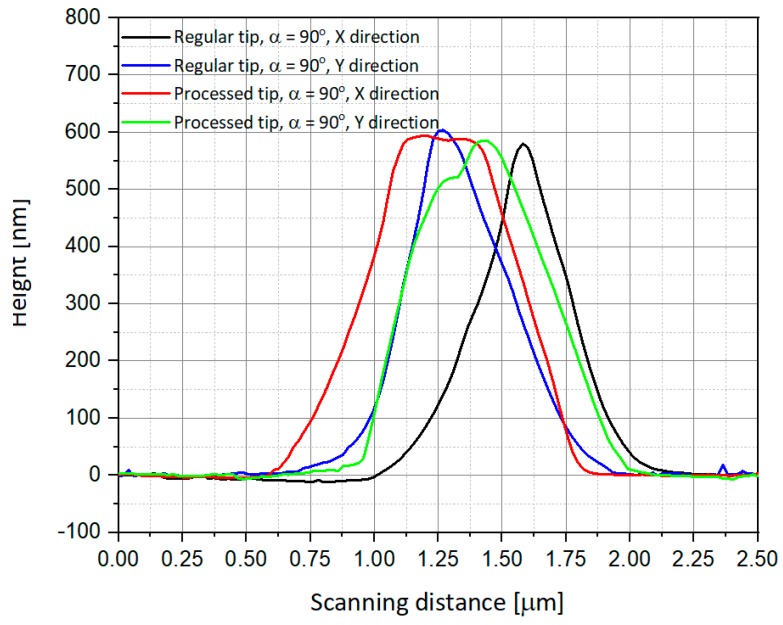
Comparison of the scanning using AFM regular and processed tips for α = 90°.

The AFM views cited above are also presented as scanning curves of α = 0° (Figure 11) and α = 90° (Figure 12). One can observe that the height of the measured tips is well-conserved in all cases, and there is almost no difference between regular and processed tips. The main difference occurs in the scanning distance, where the width of the measured peaks is larger and shifted in the case of measurement using a processed tip. Some of the measurements also present a well-known phenomenon of multiple tips [30,31], in our case a double-tip profile due to the drilled structure of the device. 

## 4. Discussion

### 4.1. AFM Conserved Functionality of the Processed Tip

One of the main concerns in this research is to assure that after the FIB ablation and drilling of a commercial tip, in order to transform it into a drilled, light-sensitive photodetector, the reduced obtained tip will continue to behave as an AFM one, meaning that the contact surface will remain small enough to scan the sample’s surface with good resolution. As mentioned above, the FIB drilling process is performed with a Helios 600 system (FEI) using a Ga^+^ ion current of 9.7 pA for an energy of 30 keV and a spot size of about 12 nm for about 10 s. This leads to an ion dose of about 5 × 10^20^ cm^−2^, which exceeds the solubility limit of 4 × 10^19^ cm^−3^, causing the silicon lattice to collapse [32]. According to a previous study that used a similar FIB system and process, the projected range of the Ga^+^ ions is about 27 nm, while the lateral range is less than 5 nm. The damaged (amorphous) area is about 50 nm deep and 23 nm wide. From the SEM picture shown in Figure 6, the contact diameter of the exposed silicon is about 200 nm. So, after the drilling step, the intact silicon close to the outer surface is about 70 nm wide, which is more than the expected extrinsic Debye length of 13 nm estimated for our 10^17^ cm^−3^ n-type silicon substrate, which allows the photocurrent to be generated in the depletion layer.

In cases where the resolution is not good enough, multiple tips [30] on the same cantilever could also be designed. The first one could be dedicated to regular AFM scanning functionality and resolution, and the second one dedicated to the detection of the light reflected from the sample with different angles, enabling NSOM functionality.

### 4.2. NSOM Working Modes vs. AFM–NSOM Dual Mode

There is a resolution trade-off between the AFM and the NSOM capabilities in our configuration. In order to correctly address the optimization process, which includes combining both methods, some considerations are necessary. The NSOM functionality is well-known and is divided into four modes of work [33]:

- The transmission mode imaging, in which the sample is illuminated through the probe, and the light passing through the sample is collected and detected;
- The reflection mode imaging, in which the sample is illuminated through the probe, and the light reflected from the sample surface is collected and detected;
- The collection mode imaging, in which the sample is illuminated with a macroscopic light source from the top or bottom, and the probe is used to collect the light from the sample’s surface, which is also our mode of operation in dual-mode;
- And the illumination–collection mode imaging, in which the probe is used for both the illumination of the sample and for the collection of the reflected signal.

Detecting the collected light can be achieved with a wide variety of instruments: an avalanche photodiode (APD), a photomultiplier tube (PMT), a CCD, or a spectrometer. The signals obtained by these detectors are then used to create an NSOM image of the surface. The trade-off in the combined system is then decreased in the AFM resolution (since the tip is ablated), but on the other hand the resolution of the NSOM increases (since the tip’s aperture can be well below the optical wavelength). 

### 4.3. NSOM–AFM Dual-Mode of Work

**Figure 13:**
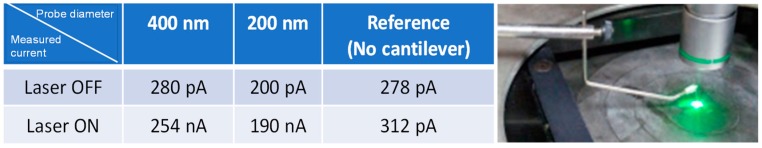
Measurement currents extracted from the dual probe while applying green laser illumination.

In this approach, the first advantage is the light collection process, which is directly performed from the surface of the sample. As a next step of our feasibility check-up, we plan to use the laser guiding spot of our AFM as an illumination source (as shown in the insert of Figure 8). This visible laser source could be modulated by an imbedded electro-optic modulator and used as a reference signal for an external lock-in amplifier. A gold wire bonded at the upper electrode will drive the small current signals (in the pA range) from the Schottky diode to the lock-in amplifier. So, as the AFM tip will scan the surface, the detected photocurrent will be converted into a NSOM image and compared to the AFM image taken in parallel. In Figure 13, we show preliminary measurements that were performed in this manner, which show that indeed there is a difference in the measured current between the vase with laser ”on” vs. “off”. Figure 13 shows an image of the measurement with the probe while being illuminated with a green laser, as well as a table showing the measured current values.

As mentioned above, the main advantages are related to the capability of our proposed NSOM–AFM dual mode system to enable both multifunctionality in one device, and also to have increased energetic collection efficiency (due to the fact that reflected light is directly converted to intensity read-out at the tip, and does not have to be coupled and guided through the collection fiber, as done in the case of conventional NSOM system).

## 5. Conclusions

In this article, a new concept of improved scanning system was presented. The system includes dual-mode NSOM–AFM capabilities, enabling multifunctionality and enhanced energetic efficiency when compared to existing tools. This win–win combined probing system enables both mechanical AFM surface scanning and topography with the light collection through a very sensitive silicon-based photodetector. This advanced system enables crossing information from mechanical and optical results during the sample scanning process, and thorough and more accurate analysis of the observed results. Preliminary results show that AFM capability was not significantly affected and there was an improvement in surface characterization for the scanning proof of concept. Since this type of combined research requires further investigation, additional experiments will be performed in the future.

## 6. Patents

A patent has been registered.

## Figures and Tables

**Table 1:** Summary table of the six-step rapid process flow.

Number and Name	Main Parameters and Legend	Process Schematics
Commercial Si-based AFM tips preparation	Type: *n* + Doping: As < 10^17^ cm^−3^	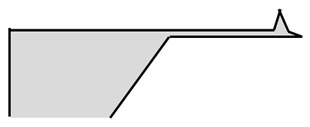
Atomic Layer Deposition	Layer: Al_2_O_3_ (red color) Thickness: 50 nm Function: Insulator	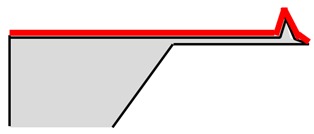
Sputtering deposition of reflective and conductive layer	Layer: Aluminum (dark gray) Thickness: 200 nm Function: Top contact (Anode)	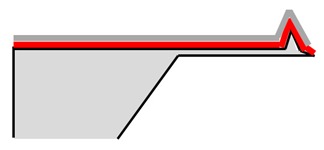
FIB ablation of the tip	Initial Si aperture diameter: 100 nm	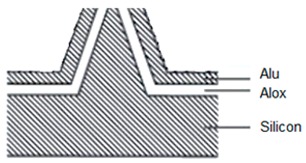
In situ FIB deposition	Layer: Platinum Thickness: 200 nm Function: Schottky contact to Si	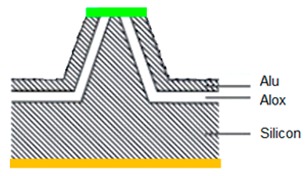
In situ FIB drilling	Detector final aperture diameter: <100 nm Depth: >200 nm